# When Does Reward Maximization Lead to Matching Law?

**DOI:** 10.1371/journal.pone.0003795

**Published:** 2008-11-24

**Authors:** Yutaka Sakai, Tomoki Fukai

**Affiliations:** 1 Brain Science Institute, Tamagawa University, Machida, Tokyo, Japan; 2 Laboratory for Neural Circuit Theory, Brain Science Institute, RIKEN, Wako, Saitama, Japan; University of Cambridge, United Kingdom

## Abstract

What kind of strategies subjects follow in various behavioral circumstances has been a central issue in decision making. In particular, which behavioral strategy, maximizing or matching, is more fundamental to animal's decision behavior has been a matter of debate. Here, we prove that any algorithm to achieve the stationary condition for maximizing the average reward should lead to matching when it ignores the dependence of the expected outcome on subject's past choices. We may term this strategy of partial reward maximization “matching strategy”. Then, this strategy is applied to the case where the subject's decision system updates the information for making a decision. Such information includes subject's past actions or sensory stimuli, and the internal storage of this information is often called “state variables”. We demonstrate that the matching strategy provides an easy way to maximize reward when combined with the exploration of the state variables that correctly represent the crucial information for reward maximization. Our results reveal for the first time how a strategy to achieve matching behavior is beneficial to reward maximization, achieving a novel insight into the relationship between maximizing and matching.

## Introduction

How do animals, including humans, determine appropriate behavioral responses when their behavioral outcomes are uncertain? Decision-making is a fundamental process of the brain for organizing behaviors, and depends crucially on how subjects have been rewarded in their past behavioral responses. Mechanism of reward-driven learning has extensively been studied theoretically and experimentally. A well-known example includes the reinforcement learning theory based on the temporal difference (TD) error algorithm[1], which is powerful enough to solve difficult problems in machine control and accounts for the basal-ganglia activity representing reward expectancy in monkeys and humans[2]–[4]. It is generally considered that subjects attempt to choose a behavioral policy that will maximize the amount of reward under a given environmental condition [5]. In addition, many algorithms in machine learning and other brain-style computations aim at reward maximization or, somewhat more generally, optimization of a given cost function.

Nevertheless, animals often exhibit matching behavior in a variety of decision-making tasks[6]–[9], even if such behavior does not necessarily maximize reward. The matching law states that the frequency of choosing an option is proportional to the amount of past reward obtained from that option[6]: *N_a_*/(*N*_1_+*N*_2_+…+*N_n_*) = *I_a_*/(*I*_1_+*N*_2_+…+*N_n_*), where *N_a_* (*a* = 1,…,*n*) represents the times option *a* has been chosen and *I_a_* the total amount of income obtained at the option. A typical example showing this law is the alternative choice task, in which subjects have to choose one from the two options that may be rewarded at different average rates. Matching and maximizing are mathematically equivalent in simple tasks[10], [11], but not in arbitrary tasks[12]–[15].

Decision-making models to reproduce the matching behavior have been proposed[9], [16], [17], and recent computational studies pointed out possible origins of matching behavior in biological neural systems[18], [19]. For instance, a recent model proposed that the matching law results from the covariance learning rule in synaptic plasticity[19]. In addition, we previously demonstrated that the matching law emerges in a class of the reinforcement learning systems including the actor-critic[20], [21], which has widely been used in engineering applications. However, whether matching and maximizing share a common computational principle and whether matching behavior is beneficial to decision making remain unclear. In this study, we propose a view that unifies matching behavior into the general computational framework of reward maximization.

## Results

We first prove that partial maximization of reward leads to matching behavior irrespective of the mathematical algorithm used for this computation. A crucial step is to define “the matching strategy” that plays a central role in the present study. We then demonstrate how the matching strategy substitutes for the maximizing strategy in a decision-making task that is difficult to solve, when matching is combined with an appropriate utilization of available information sources.

### Matching as a Sub-optimal Maximizing Strategy in Independent Choice Behaviors

The analysis is easier if we express the matching law as follows[8]:
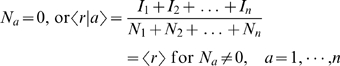
(1)where 〈*r*〉 is the average reward per choice from all options and 〈*r*|*a*〉 the average reward conditioned on choice of option *a*. We can derive the above expression from the relationship *I_a_*≅〈*r*|*a*〉*N_a_*. Thus, the matching law equalizes the expected returns on all the options that are chosen sufficiently many times. Note that the matching law should not be confused with “probability matching”[22], which states that the frequency of choosing option *a* is proportional to 〈*r*|*a*〉 rather than *I_a_*. Probability matching is typically observed in a task in which each expected return 〈*r*|*a*〉 is fixed and independent of subject's behavior (i.e., concurrent variable-ratio schedules). In such a simple task, the maximizing behavior satisfies the matching law, but not the probability matching. Hereafter, we focus on the matching law. Moreover, we consider the case where subjects make choices at fixed intervals. We can employ the discrete time steps without much loss of generality, since the framework describes a free-response task on continuous time if the interval is sufficiently short and choosing nothing is an available option.

We analyze the outcome of the decision process without specifying the detail of neural decision system. To this end, we assume a set of ‘synapses’ ***w*** = (*w*_1_, *w*_2_, …, *w_m_*) that determines the behavioral policy to make decision. These variables are often called “policy parameters” in mathematical models of decision making. Then, the probability of choosing option *a* is given as a function *p_a_*(***w***) of the synaptic weights. To ensure a smooth search for an optimal set of choice probabilities, we require that arbitrary infinitesimal changes of {*p_a_*(***w***)} allowed in the space of choice probabilities can be caused by some set of infinitesimal changes {*dw_j_*}.

With the above definitions, we can describe the average reward per choice as 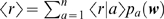. Many decision-making algorithms attempt to maximize 〈*r*〉 by modifying behavioral outputs. Whatever algorithm is used, the synaptic weights to maximize 〈*r*〉 should satisfy the stationary condition ∂〈*r*〉/∂*w_j_* = 0 for arbitrary *j*, i.e.,
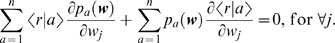
(2)


The first term contains the explicit dependence of the choice probability on *w_j_*, whereas the second term the possible change in 〈*r*|*a*〉 generated implicitly by the change in subject's behavioral policy. The conditional expectation value 〈*r*|*a*〉 is obtained by taking an average over all possible patterns of past choices in which the newest choice is option *a*. In general, the reward probability depends not only on the current choice, but also on the history of the past choices[6], [12]–[15]. In such a case, 〈*r*|*a*〉 depends on the choice probabilities that produced the past choices, and hence depends on *w_j_*.

In order to maximize reward, the brain has to explore the correct dependence of the reward probability on the past choices. It seems, however, difficult to infer this dependency correctly with little knowledge on an accurate model of the environment. In such a difficult situation, the brain may simply omit the second term in Eq. 2 in its practical attempt to maximize reward,
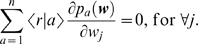
(3)


Multiplying Eq. 3 by arbitrary variations {*dw_j_*} and taking a summation over *j* gives 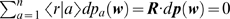, where *dp_a_*(***w***)≡Σ*_j_*(∂*p_a_*/∂*w_j_*)*dw_j_* represents the infinitesimal change caused by {*dw_j_*}, and ***R***≡(〈*r*|1〉, 〈*r*|2〉, …, 〈*r*|*n*〉) and *d* ***p***(***w***)≡(*dp*_1_(***w***), *dp*_2_(***w***), …, *dp_n_*(***w***)) are vectors in the space of multiple options. If all options have non-vanishing stationary choice probabilities, the probability changes *d* ***p***(***w***) may occur in an arbitrary direction that satisfies the probability conservation 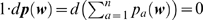, where **1**≡(1, 1, …, 1) is an *n*-dimensional identity vector. Therefore, the conditions ***R***·*d* ***p***(***w***) = 0 and **1**·*d* ***p***(***w***) = 0 can simultaneously be satisfied only by such ***R*** that is parallel to **1**. If the stationary choice probability vanishes for some option, *p_a_* = 0, we can forbid the changes in this direction (*dp_a_* = 0), and ***R*** should have identical components for all the options exhibiting non-zero choice probabilities. These results and Eq. 1 imply that the truncated stationary condition given by Eq. 3 is equivalent to the matching law.

Thus, the steady choice behavior exhibits matching when the decision system ignores the influence of subject's past choices on the expected outcome in aiming for the stationary condition of reward maximization. Hereafter, we call this suboptimal maximization strategy to achieve Eq. 3 “matching strategy”. By contrast, we call the strategy to directly solve Eq. 2 “the maximizing strategy”.

**Figure 1:**
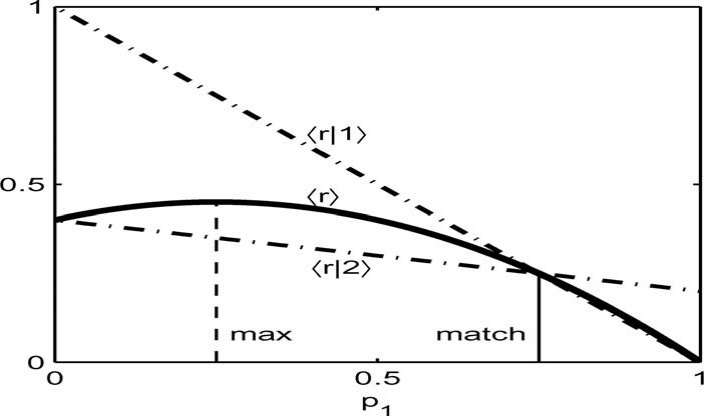
Dependences of the expectation values 〈*r*|1〉, 〈*r*|2〉 (dot-dashed lines) and 〈*r*〉 (solid curve) on *p*_1_ in a decision task with two options (Methods). The reward probability is given as a function of the current and most recent choices, but the subject makes each choice independently of the past choices. The task parameters are set as *g*_11_ = 0, *g*_21_ = 0.2, *g*_12_ = 1 and *g*_22_ = 0.4. The expectation values are given as 〈*r*|*a*〉 = *g_a_*_1_ *p*_1_+*g_a_*_2_(1−*p*_1_) and 〈*r*〉 = 〈*r*|1〉*p*_1_+〈*r*|2〉(1−*p*_1_). The matching (vertical solid line) and maximizing (vertical dashed line) choice probabilities are obtained as solutions of equations 〈*r*|1〉 = 〈*r*|2〉 and *d*〈*r*〉/*dp*_1_ = 0 respectively. The matching strategy (〈*r*〉 = 0.25) earns less than the maximizing strategy (〈*r*〉 = 0.45) in this task.

To demonstrate the above relationship between the matching and maximizing strategies, we study an alternative choice task (*n* = 2), in which the expectation value of return on each choice pattern is specified completely by the subject's current (*a_t_*) and most recent choices (*a_t_*_−1_) as 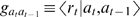 (see Methods). We consider the case where subject's current choice is independent of its past choices. Hereafter, such decision behavior is called “independent choice behavior”. Since *p*_2_(***w***) = 1−*p*_1_(***w***), the subject's decision system controls only the choice probability *p*_1_(***w***) through ***w***, and makes every choice with probability *p*_1_(***w***). Then the average return on the current choice 〈*r_t_*|*a_t_*〉 is obtained by averaging 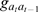 over the possible patterns *a_t_*_−1_ = 1,2 as 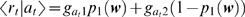, and hence depends on ***w*** through the choice probability *p*_1_(***w***). Since ∂〈*r_t_*|*a_t_*〉/∂*w_j_*≠0, the matching strategy does not maximize reward in this task. Actually, it gives 〈*r*〉 = 0.25 whereas the maximizing strategy yields 〈*r*〉 = 0.45 (Figure 1).

**Figure 2:**
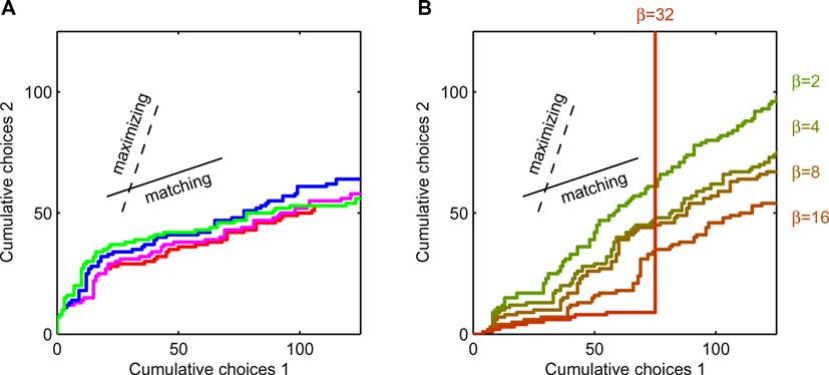
Decision behaviors generated by various decision-making systems. The horizontal and vertical axes indicate the cumulative numbers of choices given to option 1 and 2, respectively. Dashed and solid line segments indicate the slopes corresponding to the maximizing and matching choice probabilities, respectively. See Methods for details of the algorithms. (A) The actor critic (red), direct actor (magenta), local matching (blue) and melioration (green) were numerically simulated with *β* = 4. (B) The Q-leaning was simulated for *β* = 2, 4, 8, 16, and 32. At *β* = 32, the system eventually learns to choose only option 2.

The matching strategy enables us to derive a variety of learning rules that lead to matching behavior (Supporting Text S1). For instance, such a category of learning rules includes the well-known actor-critic in the reinforcement learning theory [1], [20], [21], direct actor[23], melioration[16] and local matching[9]. In particular, the actor-critic and direct actor also belong to the covariance rule[19]. We numerically solved the decision task analyzed in Figure 1 to show that all these learning algorithms generate matching behavior (Figure 2A). By contrast, indirect actor [23] does not exhibit matching in the steady behavior (Figure 2B). The indirect actor belongs to Q-learning without state variables[1] (see below for the state variables). Since Q-learning determines the choice probabilities by estimating “action values”, i.e., the expected returns on individual options, it does not show matching.

### Matching vs. Maximizing over All Possible Choice Behaviors

The quantitative analysis conducted in Figure 1 was restricted to the case where the subject generates independent choice behaviors. It was shown that the maximizing strategy earns better than the matching strategy. However, the average reward 〈*r*〉 = 0.45 achieved by the maximizing strategy in Figure 1 is not the global maximum, but is only the best one among independent choice behaviors. For instance, an alternate choice pattern of 1212…, where the current choice depends on the most recent choice, can earn better (〈*r*〉 = (*g*_12_+*g*_21_)/2 = 0.6) than the best independent choice behavior in that task. Thus, to produce a better outcome in some situation, the subject is required to make each choice depending on the past choices or other available information. Below, we investigate the relationship between the matching and maximizing strategies, taking all possible choice behaviors into account.

To make the argument as general as possible, we include the case where the subject may receive sensory signals ***σ*** *_t_* before making a choice *a_t_* at time *t*. Then, in a given task, the external and internal information available for the subject at time *t* consists of the histories of sensory signals, subject's past choices and the past returns: ***H*** *_t_* = (***σ*** *_t_*, *r_t_*_−1_, *a_t_*_−1_, ***σ*** *_t_*_−1_, *r_t_*_−2_, *a_t_*_−2_, ***σ*** *_t_*_−2_,…). A decision-making task specifies the conditional probability distribution *P*(***σ*** *_t_*_+1_, *r_t_*|*a_t_*, ***H*** *_t_*). In contrast, the general rule to determine subject's choice behavior is described by the conditional probability distribution *P*(*a_t_*|***H*** *_t_*). The problem is how to explore an optimal behavioral policy *Pˆ*(*a_t_*|***H*** *_t_*) to maximize the average reward 〈*r*〉 in a given task.

In practice, however, it is difficult to optimize the dependence of *P*(*a_t_*|***H*** *_t_*) on the whole history ***H*** *_t_*. Hence, subject's decision system may extract partial information ***s*** *_t_* from ***H*** *_t_*, and restrict the behavioral policy as
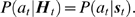
(4)


We may call the above ***s*** *_t_* “state variables”. We assume that the decision system controls the definition of state ***s*** *_t_*, ***H*** *_t_*↦***s*** *_t_*, and *P*(*a_t_*|***s*** *_t_*). In order to maximize the average reward, the decision system has to adopt an appropriate definition of state with which an optimal behavioral policy *Pˆ*(*a_t_*|***H*** *_t_*) satisfies Eq 4. It has been proved [24] that if a map ***H*** *_t_*↦***s*** *_t_* satisfies
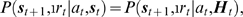
(5)for a given task, then the maximal average reward can be obtained by a behavioral policy that satisfies Eq. 4. The average reward obtained by an arbitrary choice sequence can be expressed by *P*(***s*** *_t_*_+1_, *r_t_*|*a_t_*, ***s*** *_t_*) that satisfies Eq. 5 and does not depend on the variables that are not reflected in ***s***. Therefore, state ***s*** that satisfies Eq. 5 represents crucial information about reward delivery in that task. The above theorem means that the optimal policy *Pˆ*(*a_t_*|***H*** *_t_*) depends on only the crucial information. Hereafter, we may say that a definition of state variables, ***H*** *_t_*↦***s*** *_t_*, is correct if and only if ***s*** *_t_* satisfies Eq. 5. Note that the selection of the correct definition may not be unique.

Suppose that the decision system adopts a certain definition of state variables, ***H*** *_t_*↦***s*** *_t_*. Let *p_a**s**_* = *P*(*a_t_* = *a*|***s*** *_t_* = ***s***) be the choice probability with which the decision system in state ***s*** chooses option *a*. Each state-dependent choice probability is determined as a function of the synaptic weights *p_a**s**_*(***w***). In order to explore all possible patterns of state-dependent choice probabilities smoothly, we assume that an arbitrary pattern of {*p_a**s**_*} and an arbitrary direction of infinitesimal changes {*dp_a**s**_*} allowed in the space of probabilities can be expressed by some pattern of ***w*** and some direction of infinitesimal changes *d* ***w***, respectively (see Methods).

Taking the state dependence into account, the average reward is written as 〈*r*〉 = Σ***_s_***Σ*_a_*〈*r*|*a*,***s***〉 *p_a_*_***s***_(***w***)*P*(***s***), where 〈*r*|*a*, ***s***〉 is the average reward conditioned on choice of option *a* in state ***s***, and *P*(***s***) is the distribution of the states that the subject has visited over sufficiently many decision trials with fixed {*p_a**s**_*(***w***)}. The stationary condition for reward maximization ∂〈*r*〉/∂*w_j_* = 0 is written as 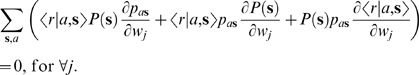
(6)


The maximizing strategy attempts to achieve Eq. 6 taking the whole dependence on ***w*** into account. In contrast, as in the previous case, the matching strategy ignores the dependence of the expected outcome of the current choice on ***w*** in aiming for the stationary condition. The outcome in the present case consists of the return *r_t_* and the next state ***s*** *_t_*_+1_. Therefore, the matching strategy ignores the dependence of *P*(***s*** *_t_*_+1_, *r_t_*|*a_t_*, ***s*** *_t_*) on ***w***, and hence ignores ∂〈*r*|*a*, ***s***〉/∂*w_j_* and ∂*P*(***s***′|*a*, ***s***)/∂*w_j_*, where *P*(***s***′|*a*, ***s***)≡*P*(***s*** *_t_*_+1_ = ***s***′|*a_t_* = *a*, ***s*** *_t_* = ***s***). By transforming the second term repetitively with the recursive relation *P*(***s***′) = Σ**_s_**_,*a*_ *P*(***s***′|*a*,***s***)*p_as_*(***w***)*P*(***s***) and by setting ∂〈*r*|*a*, ***s***〉/∂*w_j_* = ∂*P*(***s***′|*a*, ***s***)/∂*w_j_* = 0, we obtain the stationary condition of the matching strategy (Supporting Text S2): 
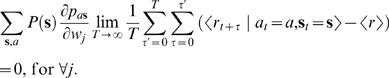
(7)


Note that the terms omitted in the matching strategy differ for different definitions of the state. Then, using Eq. 7 and the probability conservation, we can extend the matching law to the case of state-dependent choice behaviors (Supporting Text S2):
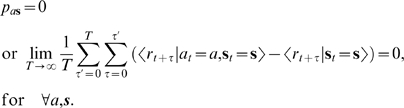
(8)


The extended matching law given as Eq. 8 depends also on the definition of the state.

**Figure 3:**
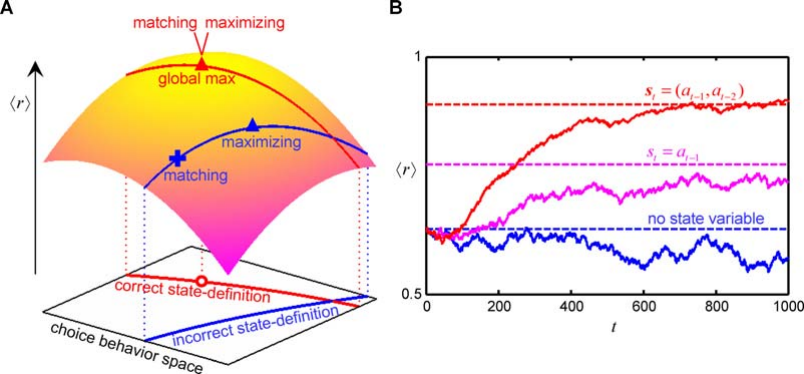
Relationship between the maximizing and matching strategies for state-dependent decision-making. (A) The performance of the matching and maximizing strategies based on correctly (red) or incorrectly (blue) defined state variables is shown schematically. (B) Actor-critic systems (Methods) were trained on a decision task in which the subject's current and most recent two choices, *a_t_*, *a_t_*_−1_ and *a_t_*_−2_, specify the reward probability according to the following task parameters: *g*_111_ = 0, *g*_211_ = 0.6, *g*_121_ = 0.9, *g*_221_ = 1, *g*_112_ = 1, *g*_212_ = 0.6, *g*_122_ = 1, and *g*_222_ = 0 (Methods). Curves and dashed lines display the local temporal averages of the rewards earned by the actor-critic systems and the best average rewards obtainable by the maximizing strategy, respectively, in three cases: no state variable (blue); an imperfect state variable *s_t_* = *a_t_*_−1_ (magenta); correct state variables *s* *_t_* = (*a_t_*_−1_, *a_t_*_−2_) (red).

We schematically illustrate the relationships between the maximizing and matching strategies with correct and incorrect definitions of the state variables (Figure 3A). The horizontal plane represents the multi-dimensional space of arbitrary choice behaviors. Defining state variables restricts the state-dependent choice behavior to a certain subspace. If state variables are correctly defined to satisfy Eq.5, the subspace (red curve) includes the optimal choice behavior (red circle). The conditional probability *P*(***s*** *_t_*_+1_, *r_t_*|*a_t_*, ***s*** *_t_*) takes a fixed value specified by the task, which is actually independent of ***w***. Therefore, the matching strategy coincides with the maximizing strategy, which indeed earns the globally maximal average reward (red triangle) unless the choice behavior is trapped by a local stationary point. In contrast, if an incorrect definition of state variables is chosen, the set of generable choice behaviors (blue curve) does not necessarily include the optimal choice behavior. Therefore, the maximizing strategy can lead to only the best choice behavior (blue triangle) within the restricted set. The conditional probability *P*(***s*** *_t_*_+1_, *r_t_*|*a_t_*, ***s*** *_t_*) depends on the past choices that are not reflected in state ***s*** *_t_*, and hence depends on ***w***. Therefore, the matching strategy (blue cross) in general deviates from the maximizing one (blue triangle).

To explain the above results, we conduct numerical simulations of a simple alternative task in which the reward probability is given as a function of the current and most recent two choices (*a_t_*, *a_t_*_−1_, *a_t_*_−2_) (see Methods). A correct definition of state variables for making choice *a_t_* is ***s*** *_t_* = (*a_t_*_−1_, *a_t_*_−2_). An actor-critic system (see Methods) operating on the correct state variables earns the globally maximal average reward (Figure 3B, red dashed line). In contrast, for an incorrectly defined state, such as *s_t_* = *a_t_*_−1_ or no state variable, the best average rewards (magenta and blue dashed lines, respectively) are smaller than the globally maximal one, and the average rewards earned by the actor-critic systems operating on the incorrect state variables (magenta and blue curves) are still smaller.

Thus, the matching strategy is as efficient as the maximizing one if they are combined with a mechanism to explore and select a correct definition of state variables. However, the matching strategy in general deviates from the maximizing one for the choice behaviors restricted by an incorrect definition of state variables.

## Discussion

How subjects decide behavioral responses based on their experience and reward expectancy is a current topic in neuroscience. In particular, which choice behavior, matching or maximizing, is more fundamental in decision making has long been debated. The relationship between matching and maximizing behaviors has been often discussed in the restricted case where every choice is independent of the past choices. For instance, Loewenstein and Seung [18] recently proved for independent choice behaviors that the maximizing behavior is achieved by synaptic learning rules that cancel out the infinite sum of the covariances between the current return and all of the current and past decision-related neural activities, and that the matching behavior appears when only the first term in the sum, i.e., the covariance between the current return and current decision-related neural activity, vanishes. This relationship corresponds to the relationship between Eqs. 2 and 3 when the choice probabilities are described as 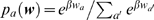 (Supporting Text S1). This study has further extended their results to derive a more general statement: any attempt to achieve the stationary condition for reward maximization results in matching behavior if it ignores the influence of the past choices on the expected outcome. This result depends on neither a specific leaning algorithm nor a specific reward schedule.

Most importantly, we have clarified the general relationship between matching and maximizing strategies among all the possible choice behaviors. We have proved that the matching strategy can lead to the optimal choice behavior when the subject's decision system correctly discovers the information sources sufficient to specify the expected outcome, and can utilize the information through state variables. Differences between the matching and maximizing strategies can arise when the decision system assigns incorrect information sources to the state variables. Our results for the first time revealed how a strategy to achieve the matching behavior is beneficial to reward maximization, and how the ignorance of the relevant information leads to the matching behavior.

The information sources relevant to the expected outcome are task-dependent. In realistic situations, the subject would have no *a priori* knowledge about the probabilistic rule of the outcomes of their behavioral responses. It seems unlikely that the brain easily identifies the relevant information sources from infinitely many combinations of the histories of past sensory inputs, returns and choices. This might explain why the matching law appears so robustly in various animal species and in various decision-making tasks as a result of ignorance of the relevant information sources. In contrast, the matching strategy with the incorrect selection of information sources may replicate various deviations from the matching behavior, such as the under/over-matching observed in various situations [25]–[28]. Our results provide a theoretical framework to investigate the deviations from matching on the basis of selected information sources. How the brain explores the relevant information sources remains open for further studies. Since this ability of the brain is what discriminates it from any existing artificial machine with human-like adaptive behavior, clarifying the underlying mechanism is an exciting challenge in neuroscience and its application to robotics.

## Methods

### Summary of assumptions

Our proof of matching law (Eq. 3) is valid for a wide class of natural learning rules, including those employing a widely-used soft-max function for choice probabilities (see below). In the following, however, we explicitly describe the assumptions necessary to make our proof mathematically rigorous. For decision-making tasks, we assumed 1) discrete time step *t* at which the subject is required to make decision, 2) a finite number of fixed options (*a* = 1, 2, …, *n*) available for the subject at every time step, and 3) a scalar amount of reward given to the subject at every time step. For the decision system, we required the following assumptions: 4) the decision system can control the definition of state ***s*** *_t_* and the state-dependent choice probabilities {*p_as_*} through a set of synapses ***w*** = (*w*_1_, *w*_2_, …, *w_m_*), 5) it adopts a definition of state ***s*** *_t_* with which the number of possible states is finite (*l*), and 6) on a certain definition of state, an arbitrary pattern of possible {*p_as_*} and an arbitrary direction of possible infinitesimal changes {*dp_as_*} can be expressed by some ***w*** and *d* ***w***, respectively. The assumption 6 requires the following condition:
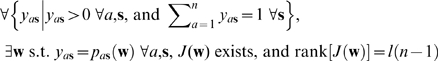
(9)where ***q***(***w***) represents the *ln*-dimensional vector function consisting of the state-dependent choice probabilities {*p_as_*(***w***)}, and *J*(***w***) is the Jacobian matrix of ***q***(***w***): *J_ij_*(***w***) = ∂*q_i_*(***w***)/∂*w_j_*. Equation 9 requires *m*≥*l*(*n*−1). Independent choice behaviors are generated in the case *l* = 1.

### Decision-making task for demonstrations

To examine the performance of the matching and maximizing strategies, we introduced a decision-making task in which reward is given (*r_t_* = 1) or not given (*r_t_* = 0) to the subject according to the probability determined by the subject's current (*a_t_*) and most recent one or two choices (*a_t_*_−1_ and *a_t_*_−2_). Each choice should be taken from one of two options (*a* = 1, 2), although it is straightforward to extend the present results to more general tasks with more than two options. The conditional expectation value of return on each choice pattern is given as a task parameter: 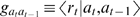 or 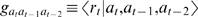. The values of these parameters are given in figure legends. For given task parameters {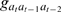}, we can calculate the maximum of the average reward 〈*r*〉 = Σ*_a_*_,*a*′,*a*″_ *g_aa_*_′*a*″_ *p_aa_*_′*a*″_ *P*(*a*′,*a*″), where *p_aa′a″_* is the conditional choice probability *p_aa_*_′*a*″_≡*P*(*a_t_* = *a*|*a_t_*_−1_ = *a*′, *a_t_*_−2_ = *a*″), and *P*(*a*′, *a*″) is the probability distribution *P*(*a*′, *a*″)≡*P*(*a_t_*_−1_ = *a*′, *a_t_*_−2_ = *a*″) obtained as a solution of equation *P*(*a*,*a*′) = Σ*_a_*_″_ *p_aa_*_′*a*″_ *P*(*a*′,*a*″). The best average rewards obtainable by the restricted choice behaviors with state-definition *s_t_*≡*a_t_*_−1_ and no state variable can be calculated by restricting *p_aa_*_′*a*″_ as *p_aa_*_′1_ = *p_aa_*_′2_ = *p_aa_*_′_ and *p_a_*_1_ = *p_a_*_2_ = *p_a_*, respectively.

### Learning rules for independent choice behaviors

Synapse-updating rules can be described by change Δ*w_j_* in *w_j_* at time *t*, *w_j_*(*t*+1) = *w_j_*(*t*)+Δ*w_j_*(*t*). Melioration[16] proposes to increase the choice probability of the option that has the largest expectation value of return. An implementation of melioration is described as *p*_1_(***w***) = *w*_0_, *p*_2_(***w***) = 1−*w*_0_, Δ*w*_0_ = *α*(*w*_1_−*w*_2_) and 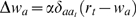, where *α* is a positive constant, and 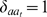 if *a_t_* = *a*, and 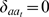 otherwise. The average returns 〈*r*|1〉 and 〈*r*|2〉 are estimated as *w*_1_ and *w*_2_, and the choice probabilities are determined by *w*_0_ updated by the estimated average returns. Local matching[9] is designed to directly achieve the matching law as 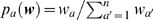 and 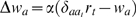. For actor-critic[1], direct actor[23] and Q-learning[1], we used a soft-max function as each choice probability: 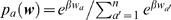, where *β* is a positive constant. Individual updating rules are described as 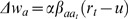 and Δ*u* = *α*(*r_t_*−*u*) (actor-critic), 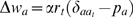 (direct actor) and 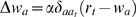 (Q-learning). The details of the algorithms and the relations to the matching strategy and the covariance rule[19] are discussed in Supporting Text S1.

### Actor-critic model with state variables

An iterative method to achieve Eq. 7 was shown in [29], [30]. Assuming a set of synapses corresponding to individual options in individual states {*w_as_*} and defining the choice probabilities in each state as 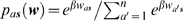, we can obtain the stochastic gradient ascent rule for Eq. 7 as 〈Δ*w_as_*〉 = *λβP*(***s***)*p_as_*(*Q_as_*−*V_s_*), where *λ* is a positive constant, and 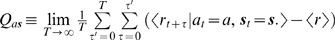 and *V* ***_s_***≡Σ*_a_Q_a_*_***s***_ *p_a_*_***s***_ represent the relative values of choosing *a* in state ***s*** (relative action-value) and of state ***s*** (relative state-value), respectively. Using the relations 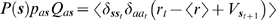 and 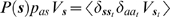, we can obtain the actor-critic model as an implementation of the matching strategy:
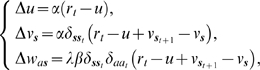
(10)where 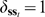 if ***s*** *_t_* = ***s***, and 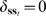 otherwise. The variable *u* estimates the average reward and the variable *ν_s_* represents the state-value of ***s*** estimated with the temporal difference (TD) error algorithm. While the actor-critic system is usually designed for maximizing a discounted sum of future rewards[1], the updating rule in Eq. 10 was derived to maximize the average reward[24], [29], [30].

### Numerical simulations

In the simulations shown in Figures 2 and 3B, model parameters were set as *α* = *λβ* = 0.05, and the initial values of all dynamical variables were set to 1. The value of *β* was set as *β* = 4 by default, while it was varied for the Q-learning simulations (Figure 2B). To show the time evolution of reward in Figure 3B, we updated the local average *y* according to Δ*y* = (*r_t_*−*y*)/200 from an initial value of 0.64, which is the average reward obtained with even choice probabilities: *p*_1_ = *p*_2_ = 0.5.

## Supporting Information

Text S1 Strategies of different learning rules. Several well-known learning algorithms are categorized into the matching, maximizing and other strategies.
(0.19 MB DOC)

Text S2 Matching strategy in state-dependent choice behaviors. The extensions of the stationary condition and the matching law are derived.
(0.19 MB DOC)
