# Supplementary material for: When Does Reward Maximization Lead to Matching Law?
Source: PLoS One. 2008 Nov 24;3(11):e3795. doi: 10.1371/journal.pone.0003795 (PMC2582656; doi:10.1371/journal.pone.0003795)
Supplement: Text S1 — Strategies of different learning rules. Several well-known learning algorithms are categorized into the matching, maximizing and other strategies. (0.19 MB DOC) [file pone.0003795.s001.doc]

**S1. Strategies of different learning rules**

**Learning rules following the matching strategy**

Several well-known learning rules exhibit matching behavior when the decision system has no state variable and each choice is independent of past choices. These learning algorithms generally obey the matching strategy. The stochastic gradient ascent designed to solve the Markov decision process[1,23] in the reinforcement learning theory presupposes that the value of expected return never changes. Both “actor critic[1]” and “direct actor[23]” are derived from the stochastic gradient ascent in the Markov decision process and exhibit matching behavior[20]. “Melioration[16]”, “local matching[9]” and “covariance rule[19]” are designed to achieve the matching law, hence providing examples of the matching strategy. Below, we show these facts explicitly.

For this purpose, we introduce a stochastic gradient ascent rule that gives a method to achieve the stationary condition given by Eq. 3 in the text: $w_{j}(t+1)=w_{j}(t)+\Delta w_{j}(t)$, $\left\langle\Delta w_{j}\right\rangle=\lambda\partial\left\langle r\right\rangle/\partial w_{j}$, where means averaging over sufficiently many trials and $\lambda$ is a positive constant. Then, the matching strategy implies

$\left\langle\Delta w_{j}\right\rangle=\lambda\sum_{a=1}^{n} \left\langle r|a\right\rangle\partial p_{a}/\partial w_{j}$ . (S1)

In the case of two options ($n=2$), we may describe the choice probabilities as $p_{1}(w)=w$ and $p_{2}(w)=1-w$. Then, we obtain $\langle\Delta w\rangle=\lambda\left(\langle r|1\rangle-\langle r|2\rangle\right)$ from Eq. (S1). This rule increases the choice probability of the option that has a larger expectation value of return, and gives an implementation of “melioration[16]”.

“Local matching[9]” estimates the average reward obtained at each option within a finite past. It defines the choice probability as the fraction of the total reward: $\Delta w_{a}=\alpha\left(\delta_{aa_{t}}r_{t}-w_{a}\right)$ and $p_{a}=w_{a}/\sum_{a'=1}^{n} w_{a'}$, where if $a_{t}=a$, and otherwise. Noting that $\langle\delta_{aa_{t}}r_{t}\rangle=p_{a}\langle r|a\rangle$ and $\left\langle w_{a}\right\rangle=\left\langle p_{a}\sum_{a'=1}^{n} w_{a'}\right\rangle\cong p_{a}\left\langle r\right\rangle$, we can derive the local matching from Eq. (S1) by setting $\lambda=\alpha w_{a}$. Note that the learning rate depends on $w_{a}$.

We can derive direct actor[23] and actor-critic by describing the choice probabilities as $p_{a}=e^{\beta w_{a}}/\sum_{a'=1}^{n} e^{\beta w_{a'}}$. Then, we can rewrite the average synaptic change as $\langle\Delta w_{a}\rangle=\lambda\beta p_{a}\left(\langle r|a\rangle-\langle r\rangle\right)$ in the stochastic gradient ascent. Noting that $p_{a}\langle r|a\rangle=\langle\delta_{aa_{t}}r_{t}\rangle$ and $p_{a}\langle r\rangle=\langle p_{a}r_{t}\rangle$, we obtain the “direct actor” $\Delta w_{a}=\alpha r_{t}(\delta_{aa_{t}}-p_{a})$, where $\alpha=\lambda\beta$. Similarly, using$p_{a}\langle r|a\rangle=\langle\delta_{aa_{t}}r_{t}\rangle p_{a}$ and $p_{a}=\langle\delta_{aa_{t}}\rangle$, and introducing a new variable $u$ for estimating $\langle r\rangle$, we can obtain the updating rule of an actor-critic system without state variables $\Delta w_{a}=\lambda\beta\delta_{aa_{t}}\left(r_{t}-u\right)$ and $\Delta v=\alpha\left(r_{t}-u\right)$.

Loewenstein and Seung[19] recently proposed a class of updating rules, called “covariance rule”, that lead to the matching law. They provided the following examples of the covariance rule: $\Delta w_{j}\propto\left(r_{t}-\langle r_{t}\rangle\right)x_{j}$, $\Delta w_{j}\propto r_{t}\left(x_{j}-\langle x_{j}\rangle\right)$, and $\Delta w_{j}\propto\left(r_{t}-\langle r_{t}\rangle\right)\left(x_{j}-\langle x_{j}\rangle\right)$. Here, $x_{j}$ represents some measure for pre- or post-synaptic activities at the *j*-th synapse. More precisely, $x_{j}$ should be correlated with the current choice, but not explicitly with the current reward. In all of the three types, the long-term average of the weight change is given as $\langle\Delta w_{j}\rangle\propto\langle r_{t}x_{j}\rangle-\langle r_{t}\rangle\langle x_{j}\rangle$. If we set $x_{a}$ to , the first and second examples of the covariance rule coincides with the actor-critic and direct actor, respectively. All the decision systems used here had no state variable.

**Learning rules following the maximizing strategy**

Examples of the maximizing strategy can be seen in the stochastic gradient ascent to solve the partially observable Markov decision process. In particular, it was recently claimed that covariance learning can maximize reward in a special case, that is, if the covariance between the current reward and an infinite sum of all past neural activities vanishes[19],

$\left\langle\Delta w_{j}\right\rangle=\lambda\sum_{\tau=0}^{\infty} \left(\left\langle r_{t}x_{j}(t-\tau)\right\rangle-\left\langle r_{t}\right\rangle\left\langle x_{j}(t-\tau)\right\rangle\right)=0$. (S2)

The matching behavior is achieved when only the first term of the infinite sum vanishes. This claim corresponds to the relationship between Eqs. 2 and 3 in the text if the choice probability function is described as $p_{a}(\mathbf{w})=e^{\beta w_{a}}/\sum_{a'} e^{\beta w_{a'}}$, since the stationary condition of the matching strategy (Eq. 3 in the text) is written as $\beta\left(\langle r_{t}\delta_{aa_{t}}\rangle-\langle r_{t}\rangle\langle\delta_{aa_{t}}\rangle\right)=0$ for $j=a$, which can be transformed into an equivalent form $\langle r_{t}(A\delta_{aa_{t}}+B)\rangle-\langle r_{t}\rangle\langle A\delta_{aa_{t}}+B\rangle=0$ where $A$ and $B$ are random variables independent of $a_{t}$ and $r_{t}$, and hence $A\delta_{aa_{t}}+B$ generally expresses decision-related neural activity $x_{j}$.

**Yet another strategy for reward maximization**

Q-learning[1] belongs to another class of the reinforcement learning algorithms that does not show matching in steady behavior. While the actor-critic system estimates the values of states, Q-learning estimates the expected returns for the individual options in each state, called “action values”, to determine choice probabilities. Several variations exist in the evaluation of future return (“SARSA”, etc.), and Q-learning without state variables is called an “indirect actor” in Dayan and Abbott[23]. If state variables are absent, Q-learning, SARSA and the indirect actor give the same algorithm: $\Delta w_{a}=\alpha\delta_{aa_{t}}\left(r_{t}-w_{a}\right)$ and $p_{a}=e^{\beta w_{a}}/\sum_{a'=1}^{n} e^{\beta w_{a'}}$. This algorithm ensures that $\left\langle w_{a}\right\rangle=\left\langle r|a\right\rangle$ in the steady state, so the choice probabilities are described by the average returns as $p_{a}\cong e^{\beta\left\langle r|a\right\rangle}/\sum_{a'=1}^{n} e^{\beta\left\langle r|a'\right\rangle}$. Since the choice probability depends on the value of a parameter (i.e., $\beta$), Q-learning cannot be reformulated in the stochastic gradient ascent and does not always exhibit the matching law.

In the practical use of Q-learning and other action-value-based learning rules, $\beta$ is set to a small value during the estimation of action values, and the greedy limit $\beta\to\infty$ is taken after this exploration. This operation ensures a maximum reward when state variables are correctly defined[24]. Thus, Q-learning gives another strategy to achieve the optimal choice behavior.
