# Supplementary material for: When Does Reward Maximization Lead to Matching Law?
Source: PLoS One. 2008 Nov 24;3(11):e3795. doi: 10.1371/journal.pone.0003795 (PMC2582656; doi:10.1371/journal.pone.0003795)
Supplement: Text S2 — Matching strategy in state-dependent choice behaviors. The extensions of the stationary condition and the matching law are derived. (0.19 MB DOC) [file pone.0003795.s002.doc]

**S2. Matching strategy in state-dependent choice behaviors**

**Stationary condition of the matching strategy**

The stationary condition given as Eq. 7 in the main text was derived for the purpose of avoiding the difficulty in calculating $\partial P(\mathbf{s})/\partial w_{j}$ in Markov decision processes[29]. We provide another derivation suitable for the present framework. To transform $\partial\left\langle r\right\rangle/\partial w_{j}$, we use the recursive relation for the long-term state distribution $P(\mathbf{s}')=\sum_{a,\mathbf{s}} P(\mathbf{s}'|a,\mathbf{s})p_{a\mathbf{s}}P(\mathbf{s})$, where $P(\mathbf{s}'|a,\mathbf{s})\equiv P(\mathbf{s}_{t+1}=\mathbf{s}'|a_{t}=a,\mathbf{s}_{t}=\mathbf{s})$. By setting $\partial\left\langle r|a,\mathbf{s}\right\rangle/\partial w_{j}=0$ and $\partial P(\mathbf{s}'|a,\mathbf{s})/\partial w_{j}=0$ according to the matching strategy, we obtain

$$\begin{matrix} \frac{\partial\left\langle r\right\rangle}{\partial w_{j}}=\frac{\partial}{\partial w_{j}}\sum_{\mathbf{s},a} \left\langle r|a,\mathbf{s}\right\rangle p_{a\mathbf{s}}P(\mathbf{s})=\sum_{\mathbf{s},a} \left\langle r|a,\mathbf{s}\right\rangle\frac{\partial p_{a\mathbf{s}}}{\partial w_{j}}P(\mathbf{s})+\sum_{\mathbf{s},a} \left\langle r|a,\mathbf{s}\right\rangle p_{a\mathbf{s}}\frac{\partial P(\mathbf{s})}{\partial w_{j}} \\ =\sum_{\mathbf{s},a} \left\langle r|a,\mathbf{s}\right\rangle\frac{\partial p_{a\mathbf{s}}}{\partial w_{j}}P(\mathbf{s})+\sum_{\mathbf{s},a} \left\langle r|a,\mathbf{s}\right\rangle p_{a\mathbf{s}}\frac{\partial\sum_{\mathbf{s}',a'} P(\mathbf{s}|a',\mathbf{s}')p_{a'\mathbf{s}'}P(\mathbf{s}')}{\partial w_{j}} \\ =\sum_{\mathbf{s},a} \left\langle r|a,\mathbf{s}\right\rangle\frac{\partial p_{a\mathbf{s}}}{\partial w_{j}}P(\mathbf{s})+\sum_{\mathbf{s}',a'} \sum_{\mathbf{s},a} \left\langle r|a,\mathbf{s}\right\rangle p_{a\mathbf{s}}P(\mathbf{s}|a',\mathbf{s}')\frac{\partial p_{a'\mathbf{s}'}P(\mathbf{s}')}{\partial w_{j}} \\ =\sum_{\mathbf{s},a} \left\langle r|a,\mathbf{s}\right\rangle\frac{\partial p_{a\mathbf{s}}}{\partial w_{j}}P(\mathbf{s})+\sum_{\mathbf{s}',a'} \left\langle r_{t+1}|a',\mathbf{s}'\right\rangle\frac{\partial p_{a'\mathbf{s}'}P(\mathbf{s}')}{\partial w_{j}} \\ =\sum_{\mathbf{s},a} \left\langle r|a,\mathbf{s}\right\rangle\frac{\partial p_{a\mathbf{s}}}{\partial w_{j}}P(\mathbf{s})+\sum_{\mathbf{s}',a'} \left\langle r_{t+1}|a',\mathbf{s}'\right\rangle\frac{\partial p_{a'\mathbf{s}'}}{\partial w_{j}}P(\mathbf{s}')+\sum_{\mathbf{s}',a'} \left\langle r_{t+1}|a',\mathbf{s}'\right\rangle p_{a'\mathbf{s}'}\frac{\partial P(\mathbf{s}')}{\partial w_{j}} \\ =\sum_{\mathbf{s},a} \left(\left\langle r|a,\mathbf{s}\right\rangle+\left\langle r_{t+1}|a,\mathbf{s}\right\rangle\right)\frac{\partial p_{a\mathbf{s}}}{\partial w_{j}}P(\mathbf{s})+\sum_{\mathbf{s},a} \left\langle r_{t+1}|a',\mathbf{s}'\right\rangle p_{a\mathbf{s}}\frac{\partial P(\mathbf{s})}{\partial w_{j}}, \end{matrix}$$

where we used the simplified expression $\left\langle r_{t+\tau}|a',\mathbf{s}'\right\rangle\equiv\left\langle r_{t+\tau}|a_{t}=a',\mathbf{s}_{t}=\mathbf{s}'\right\rangle$ and the relation $\left\langle r_{t+\tau+1}|a',\mathbf{s}'\right\rangle=\sum_{a,\mathbf{s}} \left\langle r_{t+\tau}|a,\mathbf{s}\right\rangle p_{a\mathbf{s}}P(\mathbf{s}|a',\mathbf{s}')$. Transforming $\partial\left\langle r\right\rangle/\partial w_{j}$ repetitively in the same way, we obtain

$$\begin{matrix} \frac{\partial\left\langle r\right\rangle}{\partial w_{j}}=\sum_{\mathbf{s},a} \left(\sum_{\tau=0}^{\tau'} \left\langle r_{t+\tau}|a,\mathbf{s}\right\rangle\right)\frac{\partial p_{a\mathbf{s}}}{\partial w_{j}}P(\mathbf{s})+\sum_{\mathbf{s},a} \left\langle r_{t+\tau'}|a,\mathbf{s}\right\rangle p_{a\mathbf{s}}\frac{\partial P(\mathbf{s})}{\partial w_{j}} \\ =\sum_{\mathbf{s},a} \left(\sum_{\tau=0}^{\tau'} \left(\left\langle r_{t+\tau}|a,\mathbf{s}\right\rangle-\left\langle r\right\rangle\right)\right)\frac{\partial p_{a\mathbf{s}}}{\partial w_{j}}P(\mathbf{s})+\sum_{\mathbf{s},a} \left\langle r_{t+\tau'}|a,\mathbf{s}\right\rangle p_{a\mathbf{s}}\frac{\partial P(\mathbf{s})}{\partial w_{j}}, \end{matrix}$$

where we used the relation $\left\langle r\right\rangle\sum_{a} \frac{\partial p_{a\mathbf{s}}}{\partial w_{j}}=0$ derived from the probability conservation. If the infinite sum $\sum_{\tau=0}^{\infty} \left(\left\langle r_{t+\tau}|a,\mathbf{s}\right\rangle-\left\langle r\right\rangle\right)$ converges, then $\lim_{\tau\to\infty} \left\langle r_{t+\tau}|a,\mathbf{s}\right\rangle=\left\langle r\right\rangle$ and we can simplify $\partial\left\langle r\right\rangle/\partial w_{j}$ by taking the limit: $\tau'\to\infty$. However, the infinite sum may oscillate. In order to avoid oscillations in the individual terms, we take the average over $\tau'=0,1,\cdots$ to obtain

$$\begin{matrix} \frac{\partial\left\langle r\right\rangle}{\partial w_{j}}=\lim_{T\to\infty} \frac{1}{T}\sum_{\tau'=0}^{T-1} \left(\sum_{\mathbf{s},a} \left(\sum_{\tau=0}^{\tau'} \left(\left\langle r_{t+\tau}|a,\mathbf{s}\right\rangle-\left\langle r\right\rangle\right)\right)\frac{\partial p_{a\mathbf{s}}}{\partial w_{j}}P(\mathbf{s})+\sum_{\mathbf{s},a} \left\langle r_{t+\tau'}|a,\mathbf{s}\right\rangle p_{a\mathbf{s}}\frac{\partial P(\mathbf{s})}{\partial w_{j}}\right) \\ =\sum_{\mathbf{s},a} \lim_{T\to\infty} \frac{1}{T}\sum_{\tau'=0}^{T-1} \sum_{\tau=0}^{\tau'} \left(\left\langle r_{t+\tau}|a,\mathbf{s}\right\rangle-\left\langle r\right\rangle\right)\frac{\partial p_{a\mathbf{s}}}{\partial w_{j}}P(\mathbf{s})+\sum_{s,a} \lim_{T\to\infty} \frac{1}{T}\sum_{\tau'=0}^{T-1} \left\langle r_{t+\tau'}|a,\mathbf{s}\right\rangle p_{a\mathbf{s}}\frac{\partial P(\mathbf{s})}{\partial w_{j}}. \end{matrix}$$

Noting that $\lim_{T\to\infty} \frac{1}{T}\sum_{\tau=0}^{T-1} \left\langle r_{t+\tau}|a,\mathbf{s}\right\rangle=\left\langle\left.\lim_{T\to\infty} \frac{1}{T}\sum_{\tau=0}^{T-1} r_{t+\tau}\right|a,\mathbf{s}\right\rangle=\left\langle\left.\left\langle r\right\rangle\right|a,\mathbf{s}\right\rangle=\left\langle r\right\rangle$, we obtain

$$\begin{matrix} \frac{\partial\left\langle r\right\rangle}{\partial w_{j}}=\sum_{\mathbf{s},a} \lim_{T\to\infty} \frac{1}{T}\sum_{\tau'=0}^{T-1} \sum_{\tau=0}^{\tau'} \left(\left\langle r_{t+\tau}|a,\mathbf{s}\right\rangle-\left\langle r\right\rangle\right)\frac{\partial p_{a\mathbf{s}}}{\partial w_{j}}P(\mathbf{s})+\left\langle r\right\rangle\sum_{\mathbf{s},a} p_{a\mathbf{s}}\frac{\partial P(\mathbf{s})}{\partial w_{j}} \\ =\sum_{\mathbf{s},a} \lim_{T\to\infty} \frac{1}{T}\sum_{\tau'=0}^{T-1} \sum_{\tau=0}^{\tau'} \left(\left\langle r_{t+\tau}|a,\mathbf{s}\right\rangle-\left\langle r\right\rangle\right)\frac{\partial p_{a\mathbf{s}}}{\partial w_{j}}P(\mathbf{s})+\left\langle r\right\rangle\sum_{\mathbf{s}} \frac{\partial P(\mathbf{s})}{\partial w_{j}} \\ =\sum_{\mathbf{s},a} \lim_{T\to\infty} \frac{1}{T}\sum_{\tau'=0}^{T-1} \sum_{\tau=0}^{\tau'} \left(\left\langle r_{t+\tau}|a,\mathbf{s}\right\rangle-\left\langle r\right\rangle\right)\frac{\partial p_{a\mathbf{s}}}{\partial w_{j}}P(\mathbf{s}), \end{matrix}$$

which coincides with the right hand side of Eq. 7 in the main text.

**Extension of the matching law**

Using vectors $d\mathbf{p}_{\mathbf{s}}=\left(dp_{1\mathbf{s}},dp_{2\mathbf{s}},\cdots,dp_{n\mathbf{s}}\right)$ and $\mathbf{Q}_{\mathbf{s}}=\left(Q_{1\mathbf{s}},Q_{2\mathbf{s}},\cdots,Q_{n\mathbf{s}}\right)$, where $Q_{a\mathbf{s}}\equiv\lim_{T\to\infty} \frac{1}{T}\sum_{\tau'=0}^{T-1} \sum_{\tau=0}^{\tau'} \left(\langle r_{t+\tau}|a,\mathbf{s}\rangle-\left\langle r\right\rangle\right)$, the stationary condition for the matching strategy (Eq. 7 in the text) is written as a vector form: $\sum_{\mathbf{s}} P(\mathbf{s})\mathbf{Q}_{\mathbf{s}}\cdot d\mathbf{p}_{\mathbf{s}}=0$. If the stationary choice probability $p_{a\mathbf{s}}\neq0$ for $\forall a$ and $\forall\mathbf{s}$, changes in the probabilities $\left\{d\mathbf{p}_{\mathbf{s}}\right\}$ are allowed to be in an arbitrary direction that satisfies $1\cdot d\mathbf{p}_{\mathbf{s}}=0$ for $\forall\mathbf{s}$ because of the assumption for the functions $\left\{p_{a\mathbf{s}}(\mathbf{w})\right\}$ (Eq. 9 in the main text). Therefore, the vector $\mathbf{Q}_{\mathbf{s}}$ should be parallel to the vector **1** for $\forall\mathbf{s}$. This implies $Q_{1\mathbf{s}}=Q_{2\mathbf{s}}=\cdots=Q_{n\mathbf{s}}$ for $\forall\mathbf{s}$. To obtain a stationary point along a boundary on which $p_{a\mathbf{s}}=0$ for some option in some state, we forbid the changes in this direction ($dp_{a\mathbf{s}}=0$), and obtain that the components of $\mathbf{Q}_{\mathbf{s}}$ should be identical for all options exhibiting non-zero choice probabilities. Using the summation $\sum_{a} Q_{a\mathbf{s}}p_{a\mathbf{s}}\equiv V_{\mathbf{s}}$, the above mentioned condition is written as “$p_{a\mathbf{s}}=0$ or $Q_{a\mathbf{s}}=V_{\mathbf{s}}$ for $\forall a,\mathbf{s}$”. Using $V_{\mathbf{s}}=\lim_{T\to\infty} \frac{1}{T}\sum_{\tau'=0}^{T-1} \sum_{\tau=0}^{\tau'} \left(\langle r_{t+\tau}|\mathbf{s}\rangle-\left\langle r\right\rangle\right)$,

$$\begin{matrix} Q_{a\mathbf{s}}-V_{\mathbf{s}}=\lim_{T\to\infty} \frac{1}{T}\sum_{\tau'=0}^{T-1} \sum_{\tau=0}^{\tau'} \left(\langle r_{t+\tau}|a,\mathbf{s}\rangle-\left\langle r\right\rangle\right)-\lim_{T\to\infty} \frac{1}{T}\sum_{\tau'=0}^{T-1} \sum_{\tau=0}^{\tau'} \left(\langle r_{t+\tau}|\mathbf{s}\rangle-\left\langle r\right\rangle\right) \\ =\lim_{T\to\infty} \frac{1}{T}\sum_{\tau'=0}^{T-1} \sum_{\tau=0}^{\tau'} \left(\langle r_{t+\tau}|a,\mathbf{s}\rangle-\left\langle r_{t+\tau}|\mathbf{s}\right\rangle\right). \end{matrix}$$

Thus, we obtain Eq. 8 in the text. The extended matching law is derived from the matching strategy that attempts to maximize the average reward $\langle r\rangle$. In contrast, if the brain’s decision system simply attempts to maximize the average reward in each state, $\left\langle r|\mathbf{s}\right\rangle=\sum_{a} \left\langle r|a,\mathbf{s}\right\rangle p_{a\mathbf{s}}(\mathbf{w})$, without taking the total average $\left\langle r\right\rangle=\sum_{\mathbf{s}} \left\langle r|\mathbf{s}\right\rangle P(\mathbf{s})$ into account, then the matching strategy leads to the normal matching law in the individual states: $p_{a\mathbf{s}}=0$ or $\left\langle r|a,\mathbf{s}\right\rangle-\left\langle r|\mathbf{s}\right\rangle=0$ for $\forall a$ and $\forall\mathbf{s}$. It is interesting to investigate which matching law, the normal one or the extended version, animals’ state-dependent choice behavior exhibits.
